# Oxytocin reduces anger bias, harm-intention recognition, and self-focus in behavioral variant frontotemporal dementia: a randomized double-blind placebo-controlled crossover trial

**DOI:** 10.3389/fnagi.2026.1735220

**Published:** 2026-01-22

**Authors:** Valentina Colonnello, Sabina Capellari, Maria Guarino, Piero Parchi, Luisa Sambati, Maddalena De Matteis, Michelangelo Stanzani-Maserati, Paolo Maria Russo, Katia Mattarozzi

**Affiliations:** 1 Department of Medical and Surgical Sciences, Alma Mater Studiorum, University of Bologna, Bologna, Italy; 2 IRCCS Istituto delle Scienze Neurologiche di Bologna, Bologna, Italy; 3 Department of Biomedical and Neuromotor Sciences, University of Bologna, Bologna, Italy; 4 Department of Psychology, “Sapienza” University of Rome, Rome, Italy

**Keywords:** cognition, dementia, emotion, neuropeptide, social behavior

## Abstract

**Objective:**

To explore the effects of oxytocin administration on social cognition and social engagement in patients with behavioral variant frontotemporal dementia.

**Methods:**

In a within-subject, double-blind, placebo-controlled, randomized crossover trial, patients with behavioral variant frontotemporal dementia completed the primary outcome measures, facial emotion and intention recognition tasks. Secondary outcomes included evaluation of drug safety and tolerability and changes in social engagement, assessed through caregiver ratings of social behavior. Exploratory outcomes included anger-bias in emotion misclassification and a LIWC-based analysis of speech during a semi-structured interview.

**Results:**

Oxytocin was safe, well-tolerated, and improved social engagement in naturalistic contexts: caregivers reported positive changes, including increases in awareness, spontaneous initiative, socio-affective engagement, and reductions in appetite/impulsivity. Exploratory analyses suggested that oxytocin reduced the tendency to misclassify fearful and sad expressions as angry and reduced the use of first-person pronouns during spontaneous speech. However, the study detected no improvements in the emotion recognition and observed decreased accuracy in recognizing harmful intentions.

**Conclusion:**

Exploratory findings suggest that oxytocin reduces threat-bias, promotes social engagement, and decreases self-focused language, while no improvements were detected on experimental measures of social cognition. The results highlight the safety and potential of innovative oxytocin-based pharmacological interventions and the value of incorporating naturalistic assessments, such as ecological social interactions and language analysis, to optimize the detection of therapeutic effects in neurodegenerative diseases. Oxytocin may hold promise for enhancing social engagement in behavioral variant frontotemporal dementia, although its effects on language use and higher-order cognitive processes require further investigation.

## 1 Introduction

Alterations in social cognition and interpersonal behavior are among the hallmarks of behavioral variant frontotemporal dementia (bvFTD), with symptom onset often occurring before the age of 60 (Piguet et al., 2011; Borroni et al., 2010). Individuals with bvFTD exhibit increasing difficulties interpreting social cues, engaging in empathic behavior, and maintaining appropriate social conduct (Lindberg et al., 2024; de Souza et al., 2022; Shany-Ur et al., 2012). They also show impairments in intention and emotion attribution (de Souza et al., 2022; Cerami et al., 2014), deficits in metacognition (Garcia-Cordero et al., 2021) high self-focused language use (Connelly et al., 2020), and difficulties in recognizing negative facial expressions (Bora et al., 2016; Fernandez-Duque and Black, 2005).

To date, no effective treatments targeting these social cognitive deficits have been identified. However, clinical trials have begun investigating oxytocin administration as potential treatment for behavioral symptoms in bvFTD (Jesso et al., 2011; Finger et al., 2015, 2018; Oliver et al., 2020). Such trials are grounded on affective neuroscience and psychobiological findings demonstrating that intranasal oxytocin increases concentrations in cerebrospinal fluid (Striepens et al., 2013) and modulates social behavior and cognition in healthy individuals (Domes et al., 2007; Kanat et al., 2015; Meyer-Lindenberg et al., 2011; Kosfeld et al., 2005). For example, in healthy individuals, a single dose of oxytocin increases covert attention to and the perceived duration of briefly presented happy facial expressions (Marsh et al., 2010; Domes et al., 2013; Colonnello et al., 2016). In addition, relevant to bvFTD, oxytocin appears to support flexible shifting between self- and other-related processing, as found in self-other recognition tasks in healthy individuals (Colonnello et al., 2013, 2016; Abu-Akel et al., 2015; Wang et al., 2023). Oxytocin has been suggested to increase the salience of social cues (Shamay-Tsoory and Abu-Akel, 2016) promote feelings of emotional confidence (Panksepp, 2009), and exert stress-reducing and anxiolytic effects (Kanat et al., 2015; Meyer-Lindenberg et al., 2011; Heinrichs and Domes, 2008). However, whether oxytocin also enhances complex social-cognitive functions remains unclear. For instance, while some studies report an increase in emotion recognition (Domes et al., 2007; Lischke et al., 2012), other studies have found no such effects (Straccia et al., 2023) or have seen them only in individuals with greater social difficulties (Feeser et al., 2015).

The potential for oxytocin to ameliorate social deficits in people with bvFTD also remains unclear. Previous trials have found that intranasal oxytocin is safe and well tolerated in people with bvFTD, with administered doses ranging from 24 to 72 IU twice daily (Finger et al., 2015). Consistent with the reported role of oxytocin in readiness for social engagement (Heinrichs and Domes, 2008), a single dose has been associated with improved caregiver-rated social interactions and reduced patients’ apathy (Finger et al., 2015). However, evidence regarding oxytocin’s effects on experimental social cognition tasks in people with bvFTD is mixed. For example, Jesso et al. (2011) reported reduced recognition of static angry faces following oxytocin administration, and Oliver et al. (2020) found improved performances on the Postural Knowledge Task, which assesses gesture recognition.

Building on this work, this study examined whether intranasal oxytocin administration, a well-established safe dose commonly used in research studies, affects behavioral symptoms in bvFTD. The primary outcome was social cognition, operationalized as the ability to recognize specific emotional expressions and intentions. Secondary outcomes assessed drug safety and tolerability and social engagement based on caregiver ratings. Exploratory outcomes included patterns of emotion-recognition errors (e.g., anger-bias) and psycholinguistic markers of spontaneous social language use speech during a semi-structured social interaction task with the experimenter, motivated by evidence of self-focused language in bvFTD (Connelly et al., 2020) and caregiver-reported improvements in patients’ social behavior following oxytocin (Finger et al., 2015).

To disentangle the effects of oxytocin on complex social cognition, we used dynamic emotion (Colonnello et al., 2019) and intention (Baez et al., 2014) recognition tasks to enhance ecological validity. Given Gressie et al.’s (2024) findings of default error response bias in emotion recognition in bvFTD, and evidence that oxytocin reduces neural reactivity to threat cues in healthy individuals (Kanat et al., 2015) and reduces recognition of angry expressions in patients with bvFTD (Marsh et al., 2010; Domes et al., 2013; Colonnello et al., 2016; Di Simplicio et al., 2009), we also explored whether oxytocin affects error patterns during emotion categorization.

We hypothesized that intranasal oxytocin would influence social cognition in tasks involving emotion and intention recognition, and social behavior, leading to a reduction in self-focused language and an increase in the use of social words, as well as improvements in caregiver-reported measures.

## 2 Methods

### 2.1 Participants

Participants were recruited between 2019 and 2022 through the Centro Disturbi Cognitivi e Demenze at the IRCCS Istituto delle Scienze Neurologiche Hospital Bellaria and Sant’Orsola Hospital, Bologna, Italy. The study was approved by the local ethical review board initially as of 20 June 2018 and was registered with the European Union Drug Regulating Authorities Clinical Trials (EudraCT: N. 2018-000143-12).

Patients were eligible to participate if they had a diagnosis of possible or probable bvFTD based on clinical assessment and standard neuropsychological, functional, and neurobehavioral tests (Rascovsky et al., 2011), scored 20 or higher on the Mini-Mental State Examination (MMSE) (Folstein et al., 1975), and had a caregiver available to remain with them in the hours following drug administration. A detailed description of inclusion and exclusion criteria is provided in the Supplementary material.

**Figure 1:**
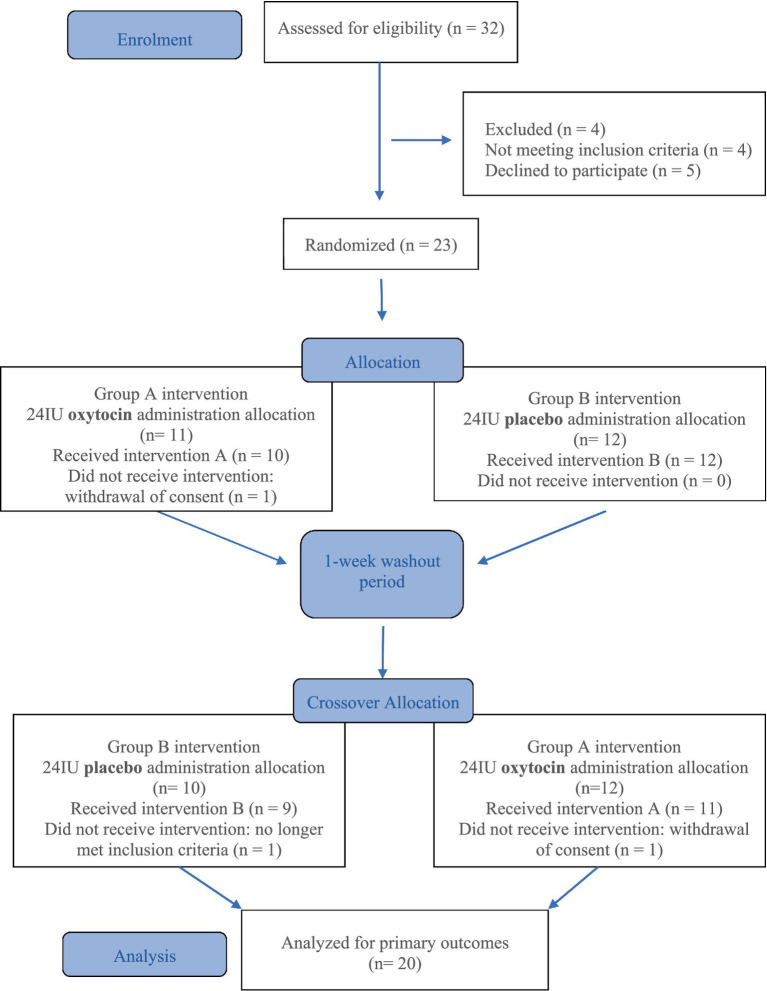
CONSORT flow diagram.

Twenty-three patients with a clinical diagnosis of possible or probable bvFTD and their caregivers were initially recruited. As shown in Figure 1, two participants dropped out and another was excluded because they did not fully meet all the inclusion criteria, resulting in a final sample of 20 participants (9 women, 11 men, mean age = 67 years, SD = 7.13).

With respect to the sample size, for the first primary outcome (change in recognizing specific emotions), a repeated-measures ANOVA with two treatments and four emotions, assuming an alpha level of 0.05, a power of 0.80, and an inter-measure correlation of *r* = 0.62 based on our previous independent study, indicated that 24 participants would be required to detect medium effects. Our final sample comprised 20 participants, which is underpowered for medium-sized effects but adequate to detect medium-to-large effects, and is acceptable given the challenges of recruiting patients with bvFTD who meet the inclusion and exclusion criteria.

A detailed summary of participants’ demographic and clinical characteristics is presented in Supplementary Table S1.

All participants and their caregivers provided written informed consent prior to participation.

### 2.2 Procedure

This study employed a within-subject, double-blind, placebo-controlled, randomized crossover design (Figure 1). To minimize fatigue associated with completing multiple tasks in a single session, testing was distributed across two consecutive days for each treatment condition. Participants received the same treatment (intranasal oxytocin or placebo) on two consecutive days at the IRCCS Hospital Bellaria, Bologna, Italy. After a 1-week washout period, they received the alternate treatment for another two consecutive days.

Intranasal administrations of oxytocin (Syntocinon spray) or placebo (identical in composition and packaging but without the active peptide) were carried out by a trained nurse. Each dose consisted of 24 IU, delivered via six puffs (three per nostril). Both the oxytocin and placebo sprays were prepared and blinded by the Pharmacy of Heidelberg University Hospital (Heidelberg, Germany) to ensure double-blind conditions.

Participants were required to refrain from eating, physical activity, caffeine, and tobacco for 2 h before testing. Following each administration, participants remained in a quiet, isolated room for approximately 30 min before beginning the experimental tasks, in line with the temporal profile of oxytocin’s behavioral effects (Heinrichs et al., 2003). During this waiting period, participants had no access to cell phones or reading materials. An experimenter monitored participants from an adjacent room to observe any side effects.

On each testing day, participants completed one of two computer-based social cognition tasks: the emotion recognition task or the intention understanding task. The order of task presentation was counterbalanced across participants and treatment phases to control for order effects. Each task was followed by a 5-min semi-structured interview with the experimenter, which was used for subsequent linguistic analysis. All testing sessions were conducted between 11:00 and 14:00.

### 2.3 Measures

#### 2.3.1 Control measures

To assess patients’ and caregivers’ expectancy effects, guesses about treatment allocation were collected immediately after the first administration in the first treatment week. Caregivers’ and patients’ guesses were recorded independently, without the other being present.

#### 2.3.2 Emotion recognition

Patients completed an emotion recognition task, previously used (Colonnello et al., 2019), based on dynamic video clips (10 s each, 25 frames/s) depicting neutral facial expressions gradually morphing into full-intensity basic emotions—happiness, anger, fear, or sadness. The stimuli were created using frontal, color images of Caucasian actors from the Karolinska Directed Faces Database (Lundqvist and Litton, 1998), with non-facial elements such as ears, hair, and background removed. Each image was digitally morphed from a neutral to a fully expressive emotional face (0 to 100% intensity) using FantaMorph© software (Abrosoft, http://www.fantamorph.com). The task included four practice trials and 48 test trials (12/emotion), with a pseudorandomized order to balance the presentation of emotions. Each trial began with a fixation cross lasting 400–600 ms.

Patients were instructed to stop the video as soon as they recognized the displayed emotion and then specify what emotion they recognized. The expression was shown at the center of the screen, and labels for the four emotions were displayed below the facial expression.

For the primary outcome of the study, the dependent measure was emotion-recognition accuracy (percentage of correct classifications for each emotion). As an exploratory analysis, we also examined anger-error bias, operationalized as the number of times same-valence negative emotions (fear and sadness) were mislabeled as an angry expression.

#### 2.3.3 Intention recognition

This task was adapted from the original empathy-for-pain paradigm previously used with patients with bvFTD (Baez et al., 2014). Participants viewed 20 video clips (each lasting 3 s, 25 frames/s) depicting harm scenarios, 10 intentional and 10 accidental. Examples of intentional harm included situations such as deliberately physically attacking someone, whereas accidental harm included scenarios such as bumping into someone while passing by. The video clips were specifically constructed to show only the actors’ body movements, with faces excluded from the frame. Two similar versions were constructed to limit possible carryover effects on the second treatment phase. The order presentation of the two versions was counterbalanced across participants.

In the original version of the task, three neutral scenarios were included. In this study, these neutral scenarios were used solely as practice trials, while the experimental focus was directed toward intentional and accidental harm scenarios.

For the primary outcome, the accuracy of intention recognition (intentional vs. accidental) was measured by the number of correct identifications of the scenario’s intention, using a dichotomous Yes/No response for each of the 20 scenarios.

As in the original version of the task, additional questions assessing the cognitive and affective components of empathy and moral judgment were included, asking participants to rate (a) the degree of the perpetrator’s harmful intent (e.g., “How bad was the intention?”), (b) empathic concern (e.g., “How sad do you feel for the person who was hurt?”), (c) personal distress (e.g., “How upset do you feel about what happened in this situation?”), (d) moral judgment (e.g., “How inappropriate was the action?”), and (e) the severity of deserved punishment (e.g., “How much penalty does this action deserve?”). All these questions were rated on a 0–100 visual scale.

#### 2.3.4 Drug safety and tolerability

Caregivers were asked to report any observed side effects occurring within 24 h after treatment administration. Caregiver reports of adverse events and side effects were collected again 1 week after the end of the second treatment week. Patients were asked about potential side effects both during the testing day and through follow-up the next day.

#### 2.3.5 Social engagement based on caregivers’ observations following drug administration

To investigate the effects of oxytocin on social behavior, caregivers were asked an open-ended question about any changes they observed in the participants’ behavior on the day of treatment and the following day (e.g., “Did you notice any changes or anything different compared to the usual in Mr./Ms. ____'s behavior. Any observation is relevant to us.”). Both the number and types of changes were recorded.

#### 2.3.6 Social engagement based on patients’ natural language use

Patients’ psycholinguistic patterns were measured during a 5 min, face-to-face, semi-structured social interaction with the experimenter. The use of a semi-structured interaction facilitated the capture of a diverse range of spontaneously generated linguistic features while allowing participants to feel comfortable and speak naturally.

This interaction took place on each testing day, resulting in two transcripts per treatment week.

A structured set of prompts guided the conversation and addressed participants’ current feelings, potential side effects of the treatment, activities performed during the waiting period, thoughts during that time, personal background and preferences, and plans or desires for the rest of the day (see the Supplementary material for the prompt template). Participants were informed that the purpose of this interaction was to monitor possible side effects and to learn more about them.

Analysis of this social interaction sample looked at verbatim transcripts of participants’ responses regarding the activities they performed during the waiting period, their thoughts during that time, their personal background and preferences, and their plans or desires for the rest of the day.

The dependent variables were derived from categories automatically identified by the Linguistic Inquiry and Word Count (LIWC) software (Boyd et al., 2022), which analyzes each text by comparing its words to those in its internal dictionary, counting all words, and calculating the percentage that falls into each LIWC predefined subcategory. The categories included the proportion of I-words (use of first-person singular pronouns; an index of self-focused language), Social words (e.g., others and social relationships), Positive and Negative tone (words reflecting emotional valence), Authenticity (i.e., honest expression), Analytic thinking (reflecting logical thinking), and Cognitive processes (words related to thinking, reasoning, and understanding) (Boyd et al., 2022).

### 2.4 Data analysis

The effectiveness of the blinding procedure was assessed by examining whether caregivers’ and patients’ guesses during the first treatment week were distributed randomly across treatment conditions, using chi-squared tests. Because three caregivers reported having no idea which treatment the patients had received, the analysis was conducted on the remaining caregivers’ responses. With respect to the computer-based tasks, preliminary analyses were conducted to assess potential order effects related to the presentation of drug conditions (oxytocin vs. placebo) and task sequence (emotion recognition vs. intention understanding). Separate repeated-measures ANOVA (analyses of variance) tests were performed with Order as a within-subject factor for each measure.

For the primary outcome, accuracy on the Emotion Recognition Task, a 2 × 4 repeated-measures ANOVA was conducted with Treatment (oxytocin, placebo) and Emotion (happiness, sadness, anger, fear) treated as within-subject factors. The dependent variable was the percentage of correctly recognized facial expressions for each emotion. For the additional primary outcome, intention recognition accuracy, the number of correctly identified accidental and intentional harm scenarios was computed. For the remaining questions on empathic and moral evaluations, mean ratings were computed across each type of scenario (accidental and intentional) separately for each treatment. Separate 2 × 2 repeated-measures ANOVAs were conducted for each dependent variable, with Treatment (oxytocin, placebo) and Scenario Type (intentional, accidental) as within-subject factors.

For secondary outcomes, drug safety and tolerability were assessed descriptively based on caregiver and patient reports of side effects following each administration. Caregiver reports on social behavior were analyzed using an inductive, data-driven thematic analysis approach (Braun and Clarke, 2006). First, two researchers (VC and MDM) independently read and re-read the interview transcripts to identify initial patterns of meaning. After coding each sentence or meaningful segment, the codes were then considered into broader categories to identify shared features across the data. The candidate themes were generated by grouping related categories, and these were reviewed and refined to ensure internal consistency and clear boundaries between themes. Inter-rater reliability for the qualitative coding of transcripts was high, with Cohen’s *κ* = 0.90 (over 90% agreement). Discrepancies between coders were discussed until consensus was reached.

With respect to the exploratory outcomes, paired-sample *t*-tests were used to compare the number of times an emotion was misclassified as threatening (angry) under the placebo and oxytocin conditions. Specifically, the percentage of times where sadness and fear (*n* = 24) were incorrectly identified as anger was calculated by dividing the number of such errors by the total number of presentations of same-valence non-angry emotions, then multiplying by 100.

For the patients’ Natural Language Use, data from one patient was excluded from the analysis because of the limited number of words produced (<50 during the interactions). Consistent with prior studies of linguistic data (Boyd et al., 2022; Chung and Pennebaker, 2008), several categories exhibited significant skewness. Thus, LIWC percentage scores were transformed to better meet the normality assumptions of ANOVA. For each category, a 2 × 2 repeated-measures ANOVA was performed on the transformed data, with Treatment and Day as within-subject factors.

For each ANOVA, Bonferroni-adjusted *post-hoc* tests were used to explore differences indicated by significant interactions, and Greenhouse–Geisser corrections were applied where the sphericity assumption was violated. For clarity, descriptive statistics (*M* ± *SD*) are reported on the original percentage scale. We did not apply additional global correction across all tasks and measures, given the limited sample size. Because anger-bias and language measures were exploratory, these findings are considered preliminary and interpreted with caution.

All analyses were performed using SPSS version 29.

## 3 Results

### 3.1 Control measures

Regarding the computer-based tasks, no significant main effects or interactions involving the factor Order of drug administration were found for any of the dependent variables (all *p*s > 0.05). The full statistical results are presented in the Supplementary material.

Caregivers’ initial guesses about drug allocation did not differ from chance, *χ*^2^(1, *N* = 17) = 1.47, *p* = 0.23, with 11 of 17 caregivers guessing correctly. Likewise, patients were unable to correctly guess their treatment allocation, *χ*^2^(1, *N* = 20) = 0.80, *p* = 0.37, with 8 of 20 patients guessing correctly.

### 3.2 Emotion recognition

The main effect of Treatment was not significant, *F*(1, 19) = 0.28, *p* = 0.60, *ηp*^2^ = 0.01.

A significant main effect of Emotion was found, *F*(3, 57) = 33.65, *p* < 0.001, *ηp*^2^ = 0.64. *Post hoc* comparisons indicated that happy facial expressions were recognized more accurately than negative facial expressions (*p* < 0.01).

With respect to the primary outcome measure, the Greenhouse–Geisser-corrected analysis (*ε* = 0.73) showed that the Treatment × Emotion interaction was not significant, *F*(2.20, 41.77) = 0.94, *p* = 0.42, *ηp*^2^ = 0.04. Supplementary material presents the means, standard deviations, and confidence intervals for all variables (Supplementary Table S2).

**Figure 2:**
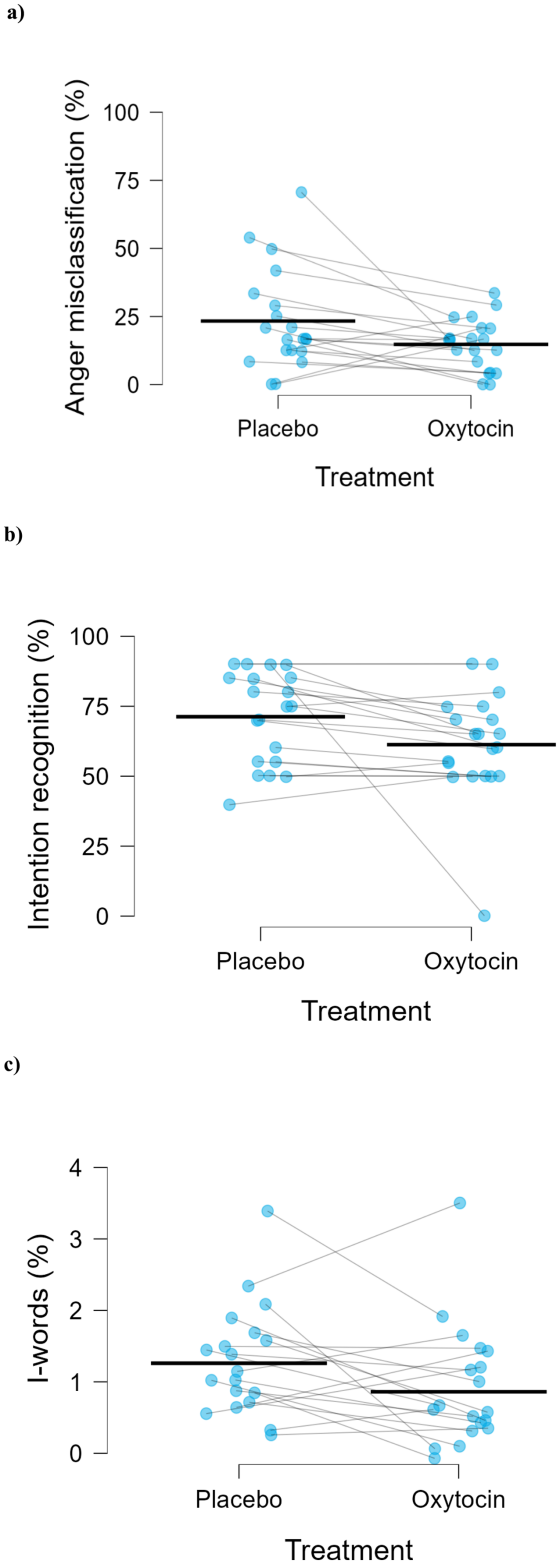
Effects of placebo and oxytocin on **(a)** misclassification of emotions as anger (*t*(19) = 2.35, *p* = 0.030, Cohen’s *d* = 0.53, *n* = 20), **(b)** the main effect of treatment on intention recognition accuracy (*F*(1,19) = 4.61, *p* = 0.045, *ηp*^2^ = 0.20, *n* = 20), and **(c)** main effect of treatment on use of I-words (*F*(1,18) = 4.63, *p* = 0.045, *ηp*^2^ = 0.20, *n* = 19). Blue dots represent individual participants, and blue lines connect paired observations across conditions. Black dashes indicate the mean for each treatment condition.

Regarding the error pattern, anger-bias was higher in the placebo (*M* = 23.33, *SD* = 18.46) than in the oxytocin condition (*M* = 14.79, *SD* = 9.41), *t*(19) = 2.35, *p* = 0.030, Cohen’s *d* = 0.53 (Figure 2a).

### 3.3 Intention recognition

As shown in Figure 2b, a significant Treatment effect was found, *F*(1, 19) = 4.61, *p* = 0.045, *ηp*^2^ = 0.20, with higher percentage accuracy in recognizing intentions under placebo (*M* = 71.25, *SD* = 16.45) compared to oxytocin (*M* = 61.25, *SD* = 19.25).

A significant main effect of Scenario was observed, with higher accuracy in recognizing intentional harm scenarios compared to accidental ones, *F*(1, 19) = 4.61, *p* = 0.045, *ηp*^2^ = 0.20.

The Treatment × Scenario interaction was not significant, *F*(1, 19) = 0.24, *p* = 0.632, *ηp*^2^ = 0.01.

A significant main effect of Scenario was found across all the remaining questions assessing the cognitive and affective components of empathy and moral judgment. Participants provided higher ratings in response to intentional harm scenarios compared to accidental harm scenarios (all *F*s > 9.33, all *p*s < 0.05). No significant main effect of Treatment emerged on any of the five measures (all *F*s < 2.49, all *p*s > 0.10), nor were there any significant Treatment × Scenario interactions (all *F*s < 2.86, all *p*s > 0.10). The full statistical results for the main effects and the interaction effect are presented in Supplementary Table S3.

### 3.4 Drug safety and tolerability

No adverse events were observed by the experimenters or reported by the patients during the study. However, caregiver observations included one report under oxytocin in which the patient was described as *“extremely gloomy, silent, and woke up several times during the night”* (Caregiver 18). Under placebo, another caregiver reported that the patient experienced *“headache and nausea [and] talked a lot at night”* (Caregiver 12). Caregiver 18 correctly guessed the drug allocation, whereas Caregiver 12 was among those who reported being unable to make a guess.

### 3.5 Caregivers’ observations following drug administration

The thematic analysis led to identification of the following domains: increased presence/awareness, autonomy/initiative, and social and affective engagement; reduced appetite/impulsivity.

Following oxytocin administration, 11 caregivers observed behavioral changes in the patients. These changes included increased awareness and engagement in daily activities, ranging from reading and writing to household and self-care tasks (reported by 7 caregivers), increased autonomy/initiative (reported by 3 caregivers), increased social and affective engagement (reported by 6 caregivers), and reduced eagerness for food (reported by 2 caregivers). However, one caregiver also reported increased irritability during the night.

Following placebo administration, only two caregivers reported changes in patient’s behavior: one caregiver reported that the patient was more active than usual, while another noted that the patient showed increased irritability.

Correct guesses were not necessarily associated with observed treatment effects. Seven caregivers who did not report any behavioral changes in the patients were able to correctly guess the drug allocation. Among the 10 caregivers who reported positive changes in patient’s behavior following oxytocin, three correctly guessed the drug allocation (Caregivers 1, 18, and 20), and two reported being unable to guess (Caregivers 11 and 12). Of the two caregivers who reported changes in patient’s behavior following placebo, one reported positive change and correctly guessed the drug allocation, while the other reported negative change and being unable to guess.

The caregivers’ comments are provided in Table 1.

**Table 1:** Main thematic categories and corresponding verbatim caregiver quotes in placebo and oxytocin conditions.

Theme	Oxytocin	Placebo
Presence/awareness	“Somewhat more present but difficult as always; at the day center they reported she was more present and with memory. At home she was … more present and attentive to what was happening, interested in the TV; interested only if she noticed something unusual in the house. Careful gaze at the stove and at the house cleaning.” (Caregiver 1) “In general, he seemed calmer, more alert, more interested, more flexible. While we were watching the news, he asked me for clarification and made a comment. Usually, he says nothing and I do not even know if he follows. Later, while watching a documentary, he explained to me what the program was, who presented it, and with more interest and enthusiasm. In general, he grasps more easily the things I say, the situation.” (Caregiver 9) “I do not know if she was given the treatment, but she has changed for the better. Much better: she reads, is present, writes, and sings.” (Caregiver 11) “Better mood, a ‘joyful’ mood; more interested in what was happening around them; calm.” (Caregiver 12) “More engaged, more alert.” (Caregiver 13) “More attentive to what was happening. Calmer.” (Caregiver 19) “More attentive, more present.” (Caregiver 20).	No change
Autonomy and initiative	“I did not see major differences, but what struck me was that she started clearing the table on her own initiative, got dressed by herself, and even took her medicine by herself. It’s a small difference, but that’s what I noticed, and it struck me. She also poured herself some water.” (Caregiver 3) “What struck me was that she chose by herself what to wear.” (Caregiver 4) “She wrote, on her own, some short thoughts in her diary.” (Caregiver 11)	No change
Social and affective engagement	“At home she was more cooperative in the afternoon.” (Caregiver 1) “Ah, and she seemed a little more curious about her daughter’s messages. And she even poured water for me.” (Caregiver 3) “A little less agitated; he asked to see an old friend.” (Caregiver 7) “In a discussion he told me, ‘You must agree too’—usually my opinion does not matter. In the same discussion he said, ‘We both need to agree’; seeing us as a unit had not happened in a long time. In the evening, when I asked him what time he wanted to eat, he said, ‘Watch the news with me. Take your time.’ This care and consideration for me had been absent for a long time. To take the bus this morning, he accepted taking a new route without arguing or insisting on the usual path.” (Caregiver 9) “I noticed a bit more collaboration.” (Caregiver 11) “More willing to listen to what I was saying.” (Caregiver 13).	“More active than usual and more compliant.” (Caregiver 6)
Appetite/impulsivity	“She ate a bit less, less voracious.” (Caregiver 4) “Less agitation about eating or smoking.” (Caregiver 11).	No change
Negative changes or no changes	“Extremely gloomy, silent, and woke up several times during the night.” (Caregiver 18) No positive or negative change (remaining 9 caregivers).	“Headache and nausea, talked a lot at night.” (Caregiver 12) No positive or negative change (remaining 18 caregivers)

### 3.6 Patients’ natural language use

A significant main effect of Treatment, *F*(1, 18) = 4.63, *p* = 0.045, *ηp*^2^ = 0.20, was found for the proportion of first-person singular pronouns (I-words), with participants using a lower percentage of I-pronouns in the oxytocin condition (*M* = 0.86%, *SD* = 0.77) than in the placebo condition (*M* = 1.26%, *SD* = 0.77) relative to their total word use (Figure 2c).

The effects of Day and Treatment × Day interaction were not significant.

No main effect of Treatment, Day, or Treatment × Day interaction were found for Social words and for the other word categories. Supplementary material presents the complete statistical findings for the main and interaction effects (Supplementary Table S4).

## 4 Discussion

Our findings provide the first preliminary evidence that acute intranasal oxytocin administration attenuates a bias toward perceiving facial threat signals and reduces self-focused natural language use (i.e., *I*-words) in patients with bvFTD. With respect to the primary outcomes, changes in recognition of specific emotions and intentions, our study did not detect an improvement following oxytocin administration in patients with bvFTD.

These findings extend the current literature on oxytocin’s effects in bvFTD by incorporating experimental tasks with dynamic social stimuli and assessing, for the first time, natural language use. Patients showed a reduced tendency to categorize fearful and sad expressions as anger following oxytocin administration compared to placebo. Though exploratory, this adds to the literature suggesting a reduced sensitivity to threat-social cues driven by oxytocin (Kanat et al., 2015), and it complements previous findings showing that oxytocin reduces the recognition of angry facial expressions in this population (Jesso et al., 2011). The pattern of findings supports the interpretation that oxytocin modulates the affective and motivational context for social interaction.

We found no changes on any of the self-reported empathy measures or moral evaluations of others’ harm intention. This resonates with previous findings indicating that oxytocin’s effects do not necessarily extend to metacognition and mentalization abilities (Straccia et al., 2023). In addition, within the limits of our sample size and experimental conditions, we observed decreased accuracy in recognizing others’ intentions based on their body movements. This finding appears to contrast Oliver et al.’s findings that oxytocin improves performance in postural task recognition (Oliver et al., 2020). Further research could investigate whether the differing effects of oxytocin are due to variations in instructions: recognizing postures among pictures in a picture-matching task versus interpreting harm intentions from video clips of body movements. Alternatively, the reduced performance observed in our task could be due to the fixed question order: the questions about the scenarios were always presented in the same order, with the question about intention shown first, immediately after the harm scenario video clip. It is possible that oxytocin interfered with the patients’ ability to shift attention from the emotional content of the video to the cognitive demands needed to accurately judge intentionality, explaining why the decline in performance emerged only for the first question. Thus, the fixed presentation order of the intentionality question may have contributed to the performance decrease observed under oxytocin.

In addition, we found that oxytocin led to changes in patients’ spontaneous language use. Exploratory psycholinguistic analysis of patients’ speech during semi-structured interviews revealed oxytocin reduced the overall proportion of first-person pronoun use, which is a marker of self-focused or egocentric attention. This change in language use is relevant given that loss of social awareness (Bozeat et al., 2000) and egocentric bias are hallmarks of bvFTD (Gressie et al., 2024), and that patients with bvFTD have been shown to use a higher proportion of I-words compared to healthy individuals (Connelly et al., 2020). Oxytocin’s ability to reduce self-focused attention during natural speech offers an interesting complement to findings that oxytocin affects the processing of self- versus other-related cues (Colonnello et al., 2013; Abu-Akel et al., 2015). That these changes occurred in a semi-structured, naturalistic context further supports the utility of measuring behavioral outcomes beyond standard cognitive tasks. This suggests the need to replicate this finding in naturalistic, everyday interactions with caregivers.

Independent caregiver reports indicated increased awareness, autonomy, and engagement in self-care or social activities in the hours following oxytocin administration in nearly half of the patients, behavioral changes that were not seen after placebo administration. Caregivers could not have anticipated the changes they reported, as the information provided in the study’s informed consent documents did not give any information about possible behavioral changes. In addition, the use of an open-ended question minimized the risk of influencing their responses. This suggests that the observed changes were unlikely to be driven by caregivers’ expectations or prompted by a predefined list of symptoms. Caregivers’ reports regarding patients’ behaviors align with previous findings of reduced apathy following oxytocin administration (Jesso et al., 2011) and point to the need for further investigation of caregiver–patient interactions following treatment.

Taken together, these findings highlight the importance of considering oxytocin’s role in affective experiential domains (Panksepp, 2009) and support the hypothesis that oxytocin’s primary effects in people with bvFTD, consistent with its evolutionarily preserved functions, are affective and motivational in nature. Our results suggest that the beneficial effects of a single dose of oxytocin in individuals with bvFTD may arise from its ability to modulate the emotional context for social interaction.

This study has limitations. The small sample size and the wide confidence intervals reduce the ability to detect subtle cognitive effects and restrict the generalizability of findings. In addition, the small sample size prevents the detection of gender differences or correlations between oxytocin’s effects and specific neuropsychological measures. Some effects reached statistical significance, but near the conventional threshold, suggesting the need to replicate the study in larger samples. Finally, the study lacks long-term follow-up and cannot exclude possible expectancy effects despite blinding. Despite these limitations, this is the first study to provide preliminary evidence that acute oxytocin administration affects emotion recognition bias and spontaneous social language use in patients with bvFTD. Importantly, no clinically significant side effects were reported by patients or caregivers following either the first or second administration, confirming that repeated oxytocin administration is safe and well-tolerated in this population. Based on our findings, optimizing the detection of therapeutic effects of intranasal oxytocin on social functioning in neurodegenerative disorders will require future research to further explore oxytocin’s effects on real-world social engagement in individuals with bvFTD.

## Data Availability

The raw data supporting the conclusions of this article will be made available by the authors, without undue reservation.
