# Supplementary material for: Oxytocin reduces anger bias, harm-intention recognition, and self-focus in behavioral variant frontotemporal dementia: a randomized double-blind placebo-controlled crossover trial
Source: Front Aging Neurosci. 2026 Jan 22;18:1735220. doi: 10.3389/fnagi.2026.1735220 (PMC12872833; doi:10.3389/fnagi.2026.1735220)
Supplement: Supplementary file 1 [file Table_1.docx]

**Supplementary material**

Inclusion and exclusion criteria

Inclusion criteria: Patients were included if they scored 20 or higher in the Mini-Mental State Examination (MMSE) and they were on stable medical treatment (i.e., treatment had not changed in the preceding two months) commonly used for bvFTD, such as acetylcholinesterase inhibitors, memantine, antidepressants, antipsychotics, mood stabilizers, or benzodiazepines. Eligible participants were between 40 and 80 years of age and included both women and men. Women had to be postmenopausal, defined as having amenorrhea for 12 months without an alternative medical cause, and not using hormonal contraception or replacement therapy; a high follicle-stimulating hormone (FSH) level in the postmenopausal range was required. Male participants with premenopausal female partners had to agree to use a double-barrier method of contraception (for example, condom plus diaphragm, or condom or diaphragm with spermicidal gel or foam) and to maintain this throughout the study. All participants were required to have normal or corrected-to-normal vision sufficient to complete the experimental tasks, be able to read and understand Italian (the language of the study instructions and consent form), and have the cognitive capacity to provide informed consent, operationalized as an MMSE score of 20 or higher and no more than 2 standard deviations below normative means on semantic fluency, Trail Making Test A-B, and Digit Span tasks. Each participant had to have an available caregiver who agreed to participate in all study sessions and complete questionnaires; a caregiver was defined as a person spending at least 8 hr/day with the patient.

Exclusion criteria included severe bvFTD (MMSE score below 20), significant speech difficulties that would interfere with assessments, or an inability to provide written informed consent. Participants were excluded if they had medical or neurological disorders of the central nervous system other than FTD or if they had clinically significant hematological, endocrine, cardiovascular, renal, hepatic, gastrointestinal, or neurological diseases—unless these were stable for at least one year and judged by the investigator not to interfere with study participation. Individuals were excluded if they had a history of myocardial infarction within the last two years or congestive heart failure, uncontrolled hypertension, bradycardia (heart rate < 50 beats/min), tachycardia (heart rate > 100 beats/min), hyponatremia (serum sodium < 135 mEq/L), or severe hepatic or renal impairment. Further exclusion criteria were a history of psychiatric disorders such as depression, bipolar disorder, or schizophrenia, use of hormonal therapy, premenopausal status in women, refusal of male participants to use a double-barrier method of contraception, alcohol or drug abuse within the past six months, ischemic cardiomyopathy, a cancer diagnosis not in remission for at least ten years, or a known allergy to food additives and preservatives used in nasal sprays (specifically E 216, E 218, and chlorobutanol hemihydrate). Finally, participants were excluded if they had used any investigational or experimental drug or device within the last 60 days or within five half-lives of the experimental drug, whichever was longer.

Semi-structured interview

A structured set of questions was used to guide the conversation, including:

How do you feel right now?/How are you feeling at this moment?

How much time were you left alone?/How long were you on your own?

What did you do while you were waiting?/What activities did you engage in while waiting?

What were you thinking about during that time?/What thoughts crossed your mind during that time?

Could you share some information about yourself?/Can you tell me a bit about yourself?

What would you like to do now, after leaving this room?/What would you like to do for the rest of the day after leaving this room?”

How long do you think we’ve been talking?/How long do you think we’ve been talking so far?”

**Table S1**
*Mean (SD) values of corrected neuropsychological test scores.*

|  | ***M*** | ***(SD)*** |
| --- | --- | --- |
| **Age (years)** | 67.14 | (7.13) |
| **Education (year)** | 10.90 | (3.64) |
| **MMSE** | 24.55 | (3.01) |
| **RAVLT-IR** | 23.27 | (10.68) |
| **RAVLT-DR** | 4.21 | (3.06) |
| **VMI** | 16.58 | (3.77) |
| **CT** | 1.55 | (1.59) |
| **CSD** | 9.42 | (2.82) |
| **SA** | 11.59 | (5.27) |
| **PVF** | 14.68 | (11.99) |
| **SVF** | 20.27 | (8.71) |
| **BDM-Index** | 0.26 | (1.25) |

Abbreviations: MMSE = Mini-Mental State Examination; RAVLT-IR = Rey Auditory Verbal Learning Test – Immediate Recall; RAVLT-DR = Rey Auditory Verbal Learning Test – Delayed Recall; VMI = Visual Memory – Immediate Recall; CT = Cancellation Test; CSD = Copy of Simple Drawings; SA = Simple Analogies; PVF = Phonemic Verbal Fluency; SVF = Semantic Verbal Fluency; BBDM = Battery for the Assessment of Mental Deterioration.

**Preliminary assessment of Order effect**

Emotion recognition accuracy.

The order of task presentation (whether the Emotion Recognition task was presented before or after the Intention Recognition task) was not significant, *F*(1,18)= 0.67; *p* = .424; pη²= .04.

The order of drug administration was not significant, *F*(1,19) = 0.17; *p* = .684; pη² = .009.

Intention recognition test

The order of task presentation (whether the Intention Recognition task was presented before or after the Emotion Recognition task) and the order of drug administration were not significant.

*Intentionality judgment accuracy*: Task order, *F*(1,19)= 0.61; *p* = .444; pη²= .031; Drug order, *F*(1,19)= 1.19; *p* = .289; pη²= .006.

*Perpetrator’s harmful intent:* Task order, *F*(1,19)= 0.19; *p* = .668; pη²= .001; Drug order, *F*(1,19)= 2.50; *p* = .13; pη²= .116.

*Empathic concern:* Task order, *F*(1,19)= 0.14; *p* = .714; pη²= .007; Drug order, *F*(1,19)= 0.11; *p* = .748; pη²= .006.

*Personal distress:* Task order, *F*(1,19)= 2.27; *p* = .148; pη²= .107; Drug order, *F*(1,19)= 0.38; *p* = .547; pη²= .019.

*Moral judgment:* Task order, *F*(1,19)= 0.52; *p* = .479; pη²= .027; Drug order, *F*(1,19)= 0.37; *p* = .850; pη²= .002.

*Severity of deserved punishment:* Task order *F*(1,19)= 0.714; *p* = .408; pη²= .004; Drug order, *F*(1,19)= 0.009; *p* = .927; pη²= .0005.

Natural language use

The order of task presentation (whether the Intention Recognition task was presented before or after the Emotion Recognition task) and the order of drug administration were not significant.

*I-words:* Task order *F*(1,18)= .83 *p* = .37; pη²= .044; Drug order, *F*(1,18)= 0.18; *p* = .677; pη²= .009.

*Positive tone*: Task order *F*(1,18)= .24 *p* = .63; pη²= .013; Drug order, *F*(1,18)= 0.50; *p* = .486; pη²= .029.

*Negative tone*: Task order *F*(1,18)= .0003 *p* = .987; pη²= .00004; Drug order, *F*(1,18)= 0.02; *p* = .878; pη²= .001.

*Social words*: Task order *F*(1,18)= .3.41 *p* = .08; pη²= .016; Drug order, *F*(1,18)= 3.27; *p* = .0.09; pη²= .154.

*Cognitive processes:* Task order *F*(1,18)= 1.77 *p* = .20; pη²= .089; Drug order, *F*(1,18)= 0.17; *p* = .681; pη²= .009.

*Analytic thinking*: Task order *F*(1,18)= .932 *p* = .347; pη²= .05; Drug order, *F*(1,18)= 0.184; *p* = .673; pη²= .01.

*Authenticity:* Task order *F*(1,18)= .005 *p* = .946; pη²= .0003; Drug order, *F*(1,18)= 0.185; *p* = .672; pη²= .18.

*Number of words*: Task order *F*(1,18)= .05 *p* = .817; pη²= .003; Drug order, *F*(1,18)= 0.46; *p* = .50; pη²= .02.

**Table S2** Mean (SD) accuracy percentages for emotion recognition, under placebo and oxytocin treatment), with 95% confidence intervals (CIs) provided below each estimate (n = 20).

| **Emotion** |  | **Placebo** | **Oxytocin** |
| --- | --- | --- | --- |
| Happiness |  | 93.33 (8.80)  89.21-97.45 | 87.92(13.10)  81.79-94.05 |
| Anger |  | 52.67 (26.44)  39.29-64.04 | 53.75 (29.80)  39.80-67.70 |
| Fear |  | 41.25 (25.00)  29.55-52.95 | 47.08 (24.22)  35.75-58.42 |
| Sadness |  | 52.50 (22.80)  41.83-63.17 | 55.75 (21.20)  43.83-63.67 |
|  |  |  |  |

**Table S3**

Mean (SD) for accuracy and ratings in intentional and accidental scenarios, under placebo and oxytocin treatment), with 95% confidence intervals (CIs) provided below each estimate (n = 20).

|  |  |  |  |  |  |  |
| --- | --- | --- | --- | --- | --- | --- |
|  |  | **Intentional scenario**  **Placebo** | **Accidental scenario**  **Placebo** | **Intentional scenario**  **Oxytocin** | **Accidental**  **Scenario**  **Oxytocin** | |
|  | Intentionality judgment accuracy | 82.5 (19.70)  73.28-91.72 | 60 (32.93)  44.58-75.41 | 75 (32.53)  59.78-90.22 | 47.5 (32.42)  32.33-62.67 | |
|  | Perpetrator’s harmful intent | 70.22 (14.54)  63.42-77.03 | 51.85 (21.76)  41.67-62.03 | 72.26 (16.74)  64.42-80.08 | 58.17 (21.26)  48.22-68.12 | |
|  | Empathic concern | 69.56 (14.80)  62.63-76.48 | 60.55 (15.40)  53.34-67.75 | 68.935 (16.81)  61.06-76.80 | 64.18 (18.48)  55.53-72.83 | |
|  | Personal distress | 66.84 (17.07)  58.85-74.82 | 60.92 (14.64)  54.07-67.77 | 66.625 (20.09)  57.23-76.02 | 61.41 (21.11)  51.53-71.29 | |
|  | Moral judgment | 74.43 (13.36)  68.17-80.67 | 61.05 (17.38)  52.91-69.17 | 73.86 (15.20)  66.75-80.97 | 65.59 (19.69)  56.37-74.81 | |
|  | Severity of deserved punishment | 74.47 (15.69)  67.12-81.81 | 56.89 (21.00)  47.06-66.71 | 70.37 (18.93)  61.51-79.23 | 57.92 (22.52)  47.38-68.46 | |
|  |  |  |  |  |  |  |

ANOVA results

*Intentionality judgment accuracy:* Treatment, *F*(1,19)= 4.61; *p* = .045; pη²= .20; Scenario, *F*(1,19) = 7.06; *p* = .012; pη² = .27; Treatment x Scenario, *F*(1,19) = .24; *p* = .632; pη² = .01.

*Perpetrator’s harmful intent:* Treatment, *F*(1,19) = 3.54; *p* = .076; pη² =.16; Scenario, *F*(1,19) = 22.17; *p* = .0001; pη² = .54; Treatment x Scenario, *F*(1,19) = 1.54; *p* = .23; pη² = .07.

*Empathic concern:* Treatment, *F*(1,19) = .414; *p* = .528, pη² = .02; Scenario, *F*(1,19) = 10.19; *p* = .005; pη² = .35; Treatment x Scenario, *F*(1,19) = 4.72; *p* = .043; pη² = .20.

*Personal distress:* Treatment, *F*(1,19) = .0025; *p* = .961; pη² = .001; Scenario, *F*(1,19) = 7.55; *p* = .013; pη² = .28; Treatment x Scenario, *F*(1,19) = .09; *p* = .767; pη² = .005*.*

*Moral judgment:* Treatment, *F*(1,19) = 1.53; *p* = .231; pη² = .07; Scenario, *F*(1,19) = 11.55; *p* = .003; pη² = .38; Treatment x Scenario, *F*(1,19) = 3.91; *p* = .063; pη² = .17.

*Severity of deserved punishment:* Treatment, *F*(1,19) = .34; *p* = .565; pη² = .02; Scenario, *F*(1,19) = 19.97; *p* = .0003; pη² = .51; Treatment x Scenario, *F*(1,19) = 3.91; *p* = .062; pη² = .17.

**Table S4** LIWC categories and word counts across two observation days, under placebo and oxytocin treatment. Values are reported as Mean (SD), with 95% confidence intervals (CIs) provided below each estimate, based on the untransformed data prior to the transformations applied to meet ANOVA normality assumptions (n = 19).

|  |  | **Placebo Day 1** | **Placebo Day 2** | **Oxytocin Day 1** | **Oxytocin Day 2** |  |
| --- | --- | --- | --- | --- | --- | --- |
|  | I-words | 1.28 (0.87)  0.87-1.67 | 1.24 (1.34)  0.63-1.85 | 0.82 (0.98)  0.41-1.31 | 0.90 (1.01)  0.42-1.34 |  |
|  | Positive -tone | 0.28 (0.42)  0.07-0.48 | 0.22 (0.30)  0.07-0.36 | 0.29 (0.41)  0.09-0.49 | 0.45 (1.4)  0.21-1.13 |  |
|  | Negative-tone | 0.15 (0.23)  0.04-0.27 | 0.33 (0.48)  0.09-0.58 | 0.16 (0.25)  0.04-0.28 | 0.23 (0.29)  0.09-0.38 |  |
|  | Social words | 0.65 (0.44)  0.45-0.86 | 0.66 (0.52)  0.42-0.90 | 0.60 (0.76)  0.25-0.96 | 0.99 (1.18)  0.43-1.54 |  |
|  | Cognitive processes | 0.48 (0.65)  0.17-0.78 | 0.75 (0.60)  0.47-1.02 | 0.54 (0.54)  0.29-0.79 | 0.52 (0.53)  0.27-0.77 |  |
|  | Analytic thinking | 88.64 (6.14) | 84.08 (20.38) | 91.43 (2.57) | 91.49 (2.50) |  |
|  | Authenticity | 85.47-91.51  0.55(0.35)  0.39-0.71 | 74.03-94-14  0.76 (0.85)  0.36-1.16 | 90.17-92.69  0.45 (0.26)  0.33-0.57 | 90.27-92.74  0.66 (0.67)  0.35-0.98 |  |
|  | Number of words | 284.32 (241.58)  167.88-400.75 | 295.16 (245.74)  176.72-413.60 | 304.58 (245.04)  186.47-422.68 | 231.79 (191.06)  139.70-323.88 |  |
|  |  |  |  |  |  |  |

ANOVA results. The analyses reported were performed on transformed data to ensure that the normality assumptions of ANOVA were met.

*I-words:* Treatment, *F*(1,18)= 4.63; *p* = .045; pη²= .20; Day, *F*(1,18) = 0.21; *p* = .655; pη² = .01; Treatment x Day, F(1,18) = .57; *p* = .458; pη² = .003.

*Positive-tone*: Treatment, *F*(1,18) = 0.358; *p* = .557; pη² =.02; Day, *F*(1,18) = 0.062; *p* = .807; pη² = .003; Treatment x Day, *F*(1,18) = 0.03; *p* = .86; pη² = .001.

*Negative-tone*: Treatment, *F*(1,18) = .134; *p* = .719, pη² = .007; Day, *F*(1,18) = 2.79; *p* = .112; pη² = .134; Treatment x Day, *F*(1,18) = 0.30; *p* = .589; pη² = .01.

*Social words*: Treatment, *F*(1,18) = .025; *p* = .875; pη² = .001; Day, *F*(1,18) = 2.245; *p* = .151; pη² = .11; Treatment x Day, *F*(1,18) = 3.26; *p* = .088; pη² = .153.

*Cognitive processes*: Treatment, *F*(1,18) = 0.647; *p* = .432; pη² = .03; Day, *F*(1,18) = 0.739; *p* = .401; pη² = .04; Treatment x Day, *F*(1,18) = 0.957; *p* = .341; pη² = .05.

*Analytic thinking*: Treatment, *F*(1,18) = 1.672; *p* = .212; pη² = .085; Day, *F*(1,18) = 0.922; *p* = .345; pη² = .05; Treatment x Day, *F*(1,18) = 1.008; *p* = .329; pη² = .05.

*Authenticity*: Treatment, *F*(1,18) = 0.933; *p* = .347; pη² = .05; Day, *F*(1,18) = 1.533; *p* = .231; pη² = .08; Treatment x Day, *F*(1,18) = 0.265; *p* = .612; pη² = .01.

*Number of words*: Treatment, *F*(1,18) = .441; *p* = .515; pη² = .024; Day, 1.040; *p* = .321; pη² = .055; Treatment x Day, *F*(1,18) = 3.042; *p* = .098; pη² = .145.
